# Zopiclone versus placebo for short-term treatment of insomnia in patients with advanced cancer: study protocol for a double-blind, randomized, placebo-controlled, clinical multicenter trial

**DOI:** 10.1186/s13063-018-3088-3

**Published:** 2018-12-27

**Authors:** Gunnhild Jakobsen, Morten Engstrøm, Ørnulf Paulsen, Karin Sjue, Sunil X. Raj, Morten Thronæs, Marianne Jensen Hjermstad, Stein Kaasa, Peter Fayers, Pål Klepstad

**Affiliations:** 1 European Palliative Care Research Centre (PRC), Department of Clinical and Molecular Medicine, Faculty of Medicine and Health Sciences, NTNU, Norwegian University of Science and Technology and St. Olavs hospital, Trondheim University Hospital, Trondheim, Norway; 2 Cancer Clinic, St. Olavs hospital, Trondheim University Hospital, Trondheim, Norway; 3 Department of Neuromedicine and Movement Science, Norwegian University of Science and Technology, Trondheim, Norway; 4 Department of Neurology and Clinical Neurophysiology, St. Olavs hospital, Trondheim, Norway; 5 Palliative Care Unit, Telemark Hospital Trust, Skien, Norway; 6 Department of Oncology, Vestfold Hospital Trust, Tønsberg, Norway; 7 European Palliative Care Research Centre (PRC), Department of Oncology, Oslo University Hospital, and Institute of Clinical Medicine, University of Oslo, Oslo, Norway; 8 Division of Applied Health Sciences, University of Aberdeen, Aberdeen, UK; 9 Department of Anaesthesiology and Intensive Care Medicine, St. Olavs hospital, Trondheim University Hospital, Trondheim, Norway; 10 Department of Circulation and Medical Imaging, Faculty of Medicine and Health Sciences, Norwegian University of Science and Technology NTNU, Trondheim, Norway

**Keywords:** Advanced cancer, Insomnia, Pharmacological treatment, Randomized controlled trial

## Abstract

**Background:**

Despite the high prevalence of insomnia in patients with advanced cancer, there are no randomized controlled trials on pharmacological interventions for insomnia in this group of patients. A variety of pharmacological agents is recommended to manage sleep disturbance for insomnia in the general population, but their efficacy and safety in adults with advanced cancer are not established. Thus, there is a need to evaluate the effectiveness of medications for insomnia in order to improve the evidence in patients with advanced cancer. One of the most used sleep medications at present in patients with cancer is zopiclone.

**Methods:**

This is a randomized, double-blind, placebo-controlled, parallel-group, multicenter trial. A total of 100 patients with metastatic cancer who report insomnia will be randomly allocated to zopiclone or placebo. The treatment duration with zopiclone/placebo is 6 consecutive nights. The primary endpoint is patient-reported sleep quality during the final study night (night 6) assessed on a numerical rating scale of 0–10, where 0 = Best sleep and 10 = Worst possible sleep. Secondary endpoints include the mean patient-reported total sleep time and sleep onset latency during the final study night (night 6).

**Discussion:**

Results from this study on treatment of insomnia in advanced cancer will contribute to clinical decision-making and improve the treatment of sleep disturbance in this patient cohort.

**Trial registration:**

ClinicalTrials.gov, NCT02807922. Registered on 21 June 2016.

**Electronic supplementary material:**

The online version of this article (10.1186/s13063-018-3088-3) contains supplementary material, which is available to authorized users.

## Background

Sleep disturbance is frequent in patients with advanced cancer and might impair the tolerance of other symptoms and reduce quality of life [1, 2]. An observational study in different settings of palliative care found that sleep disturbance was reported by 61% of the cancer patients [3]. This prevalence corresponds with results from another multicenter study where 78% of the patients with advanced cancer experienced poor sleep quality [4]. Insomnia is the most prevalent sleep disorder both in the general population and among patients with cancer [5]. The prevalence of insomnia in the general population, when defined according to formal diagnostic systems, is approximately 10% [6, 7]. The prevalence of insomnia in patients with cancer is reported to be threefold higher [8]. Insomnia in the context of cancer is characterized as difficulty initiating sleep and/or difficulty maintaining sleep, which impairs daytime functioning [9].

Sleep quality in patients with advanced cancer might be influenced by several factors such as pain, fatigue, and anxiety, worries about the future, little daytime activity, interrupted sleep because of procedures, a large number of medications, and systemic inflammation [1, 10–12]. In palliative care cancer populations, where life expectancy is limited, the goal of symptom relief usually overrides the concerns of long-term side effects [13]. However, because of polypharmacy and advanced cancer disease, the patients are more vulnerable to potential short-term side effects. Thus, results from studies on pharmacological treatment for insomnia in non-cancer populations are not necessarily applicable to patients with advanced cancer. A careful selection of sleep medications that benefit advanced cancer patients is therefore important.

A combined step-wise pharmacological and non-pharmacological approach is recommended in the treatment of insomnia in cancer patients [14]. In cases where sleep disturbance is due to the cancer or its treatment, the first step is to remove the causative condition if possible (e.g., pain, dyspnea, anxiety) [15]. The second step should include non-pharmacological sleep interventions with cognitive behavioral therapy [16]. Cognitive behavioral therapy for insomnia (CBT-I) usually consists of psychoeducation/sleep hygiene, relaxation training, stimulus control therapy, and cognitive therapy [17]. In a European guideline, CBT-I is recommended as the first line treatment for chronic insomnia in adults of any age [18]. A randomized controlled trial (RCT) supported the efficacy of CBT-I for chronic insomnia associated with cancer [19]. However, a major limitation for the routine implementation of CBT-I in cancer clinics is the lack of mental health professionals formally trained in this approach. In addition, the number of CBT sessions, usually four to eight sessions, can be too demanding for patients during cancer treatment or for patients with advanced cancer disease. Thus, when life expectancy is short, medication is recommended as the treatment of choice for insomnia because of its rapid effect [20, 21].

To manage insomnia successfully, pharmacological treatment for insomnia should reduce sleep latency, increase sleep maintenance, and improve sleep quality [22]. A variety of pharmacological agents against insomnia has been recommended for use in the general population, but their efficacy and safety in adults with cancer are not established [16]. A Cochrane Database review of clinical trials assessing the safety and efficacy of benzodiazepines or benzodiazepine receptor agonists in treating insomnia in palliative care identified no articles that met the inclusion criteria [23]. In 2013, Howell et al. reviewed the evidence base for the management of cancer-related sleep disturbance (insomnia and insomnia syndrome) in oncology practice, and identified no RCT data involving pharmacological interventions for insomnia in advanced cancer [16]. Despite the lack of evidence, several classes of sleep medications are used to treat sleep disturbance in patients with cancer [24]. In a European multicenter study, the most frequently used hypnotic drug was the non-benzodiazepine hypnotic agent zopiclone, which was used by 348 of 2282 patients with advanced cancer [24]. Therefore, we designed a double-blind, placebo-controlled, parallel-group RCT to evaluate the short-term effectiveness of zopiclone on self-reported sleep quality in patients with advanced metastatic cancer who report insomnia.

## Methods

The trial adheres to the Standard Protocol Items: Recommendations for Interventional trials (SPIRIT) checklist (see Additional file 1).

### Study design

This study is designed as a randomized, double-blind, placebo-controlled, parallel-group, multicenter, phase IV clinical trial.

### Study objectives and endpoints

The primary objective is to study the short-time effectiveness of zopiclone on patient-reported subjective sleep quality in patients with advanced cancer who report insomnia. The primary endpoint is patient-reported sleep quality during the final study night with treatment (night 6) assessed on a numerical rating scale (NRS) of 0–10, where 0 = Best sleep and 10 = Worst possible sleep. The secondary objective is to study the effectiveness of zopiclone on mean self-reported total sleep time and sleep onset latency. Table 1 summarizes the objectives and endpoints including the explorative objectives.

**Table 1:** Primary, secondary, and explorative objectives and related endpoints and assessments

	Objective	Endpoint	Assessment method
Primary	To study the short-time effectiveness of zopiclone on patient-reported subjective sleep quality	Patient-reported sleep quality the last study night (night 6)	Numerical rating scale 0–10, 0 = Best sleep, 10 = Worst possible sleep
Secondary	To study the short-time effectiveness of zopiclone on patient-reported TST	Patient-reported TST (minutes) the last study night (night 6)	Sleep diary
	To study the short-time effectiveness of zopiclone on patient-reported SOL	Patient-reported SOL (minutes) the last study night (night 6)	Sleep diary
Explorative	To study the short-time effectiveness of zopiclone on overall patient-reported sleep quality	Patient-reported overall sleep quality over a week time interval	Pittsburgh Sleep Quality Index
	To study the short-time effectiveness of zopiclone on objectively measured TST, SOL, SE, WASO, and NWAK	TST (minutes), SOL (minutes), SE (percent), WASO and NWAK during the last study night (night 6)	Actigraphy
	To study the short-time effectiveness of zopiclone on patient-reported TST, SOL, SE, WASO, and NWAK	TST (minutes), SOL (minutes), SE (percent), WASO, and NWAK during the last study night (night 6)	Sleep diary
	To study the short-time effectiveness of zopiclone on self-reported daytime sleepiness	Daytime sleepiness in the morning after night 6	Karolinska Sleepiness Scale

*TST* total sleep time, *SOL* sleep onset latency, *SE* sleep efficiency, *WASO* wake after sleep onset, *NWAK* number of awakenings

### Sample size

The primary variable used in the sample size calculation is patient-reported sleep quality on an NRS of 0–10 during night 6 (the last study night with treatment). The defined minimal clinical important difference (MCID) between the two study groups (zopiclone/placebo) is 2 for the primary outcome sleep quality, scored on the NRS. Due to the lack of relevant data for MCID for NRS on sleep quality, this number was chosen based upon NRS in cancer pain studies [25, 26]. Data obtained in recent studies suggest an expected standard deviation of 2.75 [27–33]. Using a significance level of 5%, these assumptions indicate that for 80% power 33 patients are needed in each group. For 90% power a sample size of 42 per group is required. The study will aim for a 90% power and allow for an expected number of 8 drop-outs during the study period (6 nights). The study will include 50 patients in each treatment arm.

### Eligibility criteria

Patients with advanced cancer with insomnia syndrome will be recruited by study personnel from three outpatient units at hospitals in Norway. Cancer patients are screened using the following two questions: “Do you have problems with your sleep or sleep disturbance on average for 3 or more nights a week?” “Does the problem with your sleep negatively affect your daytime functioning?” Informed consent will be obtained for all patients included in the study.

#### Inclusion criteria

The criteria for inclusion in the study are:

- Histologically verified malignant disease
- Presence of metastatic/disseminated disease
- Presence of insomnia syndrome in the context of cancer defined as [9]:
  - ◦ Self-reported difficulty initiating sleep (greater than 30 min to sleep onset) and/or difficulty maintaining sleep (greater than 30 min nocturnal waking time) and/or waking up earlier than desired; and
  - ◦ Sleep difficulty at least 3 nights per week; and
  - ◦ Sleep difficulty that causes significant impairment of daytime functioning (the patient will be asked if sleep difficulty results in altered daytime function, i.e., feeling tired, lack of energy)
- Patient at least 18 years of age
- Ability to comply with all study procedures
- Provision of signed informed consent

#### Exclusion criteria

The following criteria are grounds for exclusion from the study:

- Ongoing treatment or previous treatment (within last 4 weeks) for more than 3 consecutive days with medications given for insomnia
- Adverse reactions to zopiclone
- History of substance abuse
- Concomitant use of rifampicin and erythromycin
- Any other contraindication listed on the summary of product characteristics of zopiclone
  - ◦ Myasthenia gravis
  - ◦ An established diagnosis of severe impairment of respiratory function
  - ◦ An established diagnosis of severe hepatic insufficiency (Child-Pugh grade B or C)
  - ◦ An established diagnosis of sleep apnea
  - ◦ Known hypersensitivity to the drug or to any component in its formulation
- Unfit for participation for any reason as judged by the investigator
- Pregnancy or lactation
- Women of reproductive age not willing or unable to employ an effective method of contraception (sterilization, using IUD or oral contraception)
- Scheduled surgery within the next week
- Needing change in scheduled opioid dose at baseline (study visit 1)
- Scheduled intravenous administration of chemotherapy during the study period (from baseline to day 8) or intravenous administration of chemotherapy during the last week
- Change in corticosteroid dose last week before baseline or planned dose change in corticosteroid dose within 7 days from baseline

### Randomization, blinding, and study monitoring

**Fig. 1:**
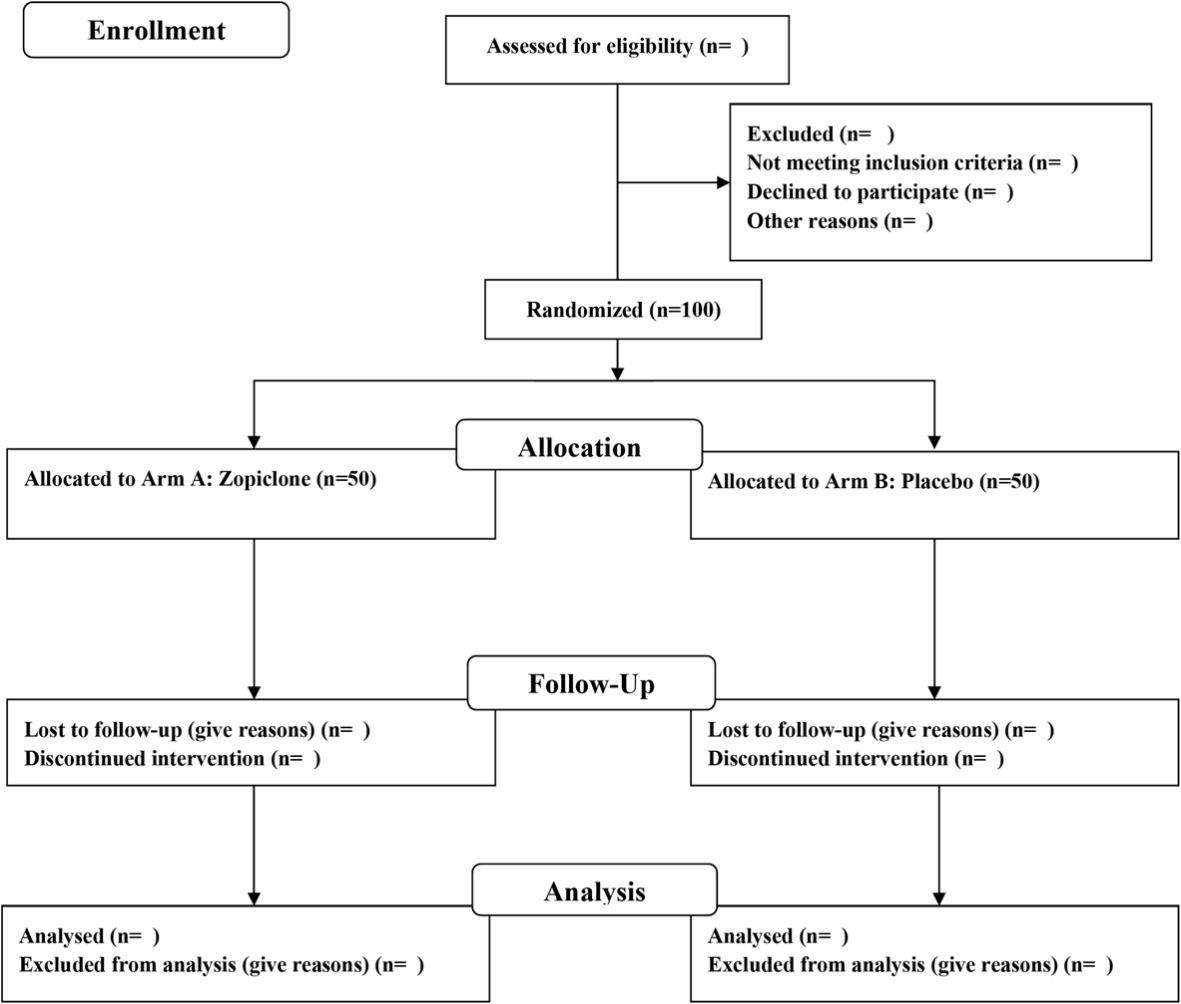
Consolidated Standards of Reporting Trials (CONSORT) flow diagram

Patients who have consented to participate in the study will be randomized using a web-based randomization system developed and administered by the Unit for Applied Clinical Research, Institute of Cancer Research and Molecular Medicine, Norwegian University of Science and Technology (NTNU), Trondheim, Norway. Patients are randomized to either the zopiclone arm (Arm A) or the placebo arm (Arm B) (Fig. 1). Randomization will be stratified based on study center. Patients are allocated with equal probabilities to treatment. The treatment is double-blind; i.e., the investigational medicinal product (IMP) is blinded for the patient, site, and study personnel by identical appearance, shape, and color as well as identical labeling and packing. In a medical emergency, the investigator will be able to break the code for an individual patient by contacting the person on call at the hospital pharmacy. The external monitor for the trial is the Unit for Applied Clinical Research, NTNU.

### Treatment

**Fig. 2:**
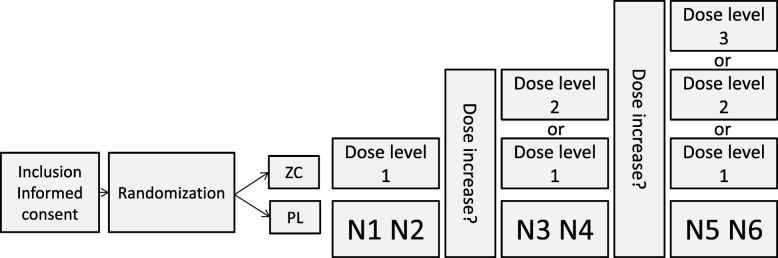
N1–N6 are 6 nights of intervention with zopiclone/placebo. The initial dose is zopiclone/placebo 3.75 mg (dose level 1). Dose level 2 is 5 mg zopiclone/placebo, and dose level 3 is 7.5 mg zopiclone/placebo

The treatment is 6 subsequent nights with zopiclone or placebo. For this study, zopiclone “Actavis” 3.75 mg, 5 mg, and 7.5 mg (active comparator) are defined as the IMP. Kragerø Tablettproduksjon AS, Kragerø, Norway will produce, blind, pack, and label the IMP. Patients will receive the IMP in custom-made capsules containing zopiclone Actavis 3.75 mg, 5 mg, or 7.5 mg or placebo administered once daily (taken in the evening 30 min before going to bed). Each patient will receive six boxes, with each box containing the allocated IMP for 2 nights. Each box is labeled with dose level according to the titration. The initial dose of zopiclone/placebo is 3.75 mg/day started after 1 night with sleep quality assessment and symptom assessment. The study personnel will evaluate the patient by phone after 2 and 4 nights of being on study medication by asking the question “Please circle the number that best describes how you feel now” on an NRS, where 0 = Best sleep and 10 = Worst possible sleep. If the NRS value is ≥4 at evaluation (Fig. 2), the dose of zopiclone/placebo is increased one dose level according to dose level 1 = 3.75 mg zopiclone/placebo; dose level 2 = 5 mg zopiclone/placebo; dose level 3 = 7.5 mg zopiclone/placebo. If NRS is < 4 at evaluation, the patient continues on the same dose level. If patients titrated to dose level 2 at night 4 experience excess sedation during the daytime, the dose can be decreased to dose level 1 for nights 5 and 6. The patients will indicate in the sleep diary whether they have taken their trial drug or not and state the reason, given by the alternatives forgotten, side effects, or other, if they omitted the trial medication. Each patient will be instructed to contact the investigator immediately should they manifest any signs or symptoms they perceive as serious. End-of-protocol therapy is defined as therapy after 6 nights with treatment. Further treatment is the responsibility of the physician in charge of patient treatment. The protocol was considered as a low-risk study, and according to Norwegian legislations, a data monitoring committee was not needed.

### Withdrawal criteria

Patients may withdraw from the trial at any time at their own request. Patients can be withdrawn from the study at the discretion of the treating physician if they have an acute complication of the cancer or the treatment, including sudden changes in their clinical condition such as cardiovascular events or sepsis.

### Data collection and statistics

The following variables will be recorded in the web-based case report form, which is administered by the Unit for Applied Clinical Research, (NTNU): date of birth, weight, height, concomitant diseases, psychiatric history, department category (i.e., palliative care unit or general oncology ward), principal cancer diagnosis (International Classification of Diseases (ICD-10)), date of the principal cancer diagnosis, site of metastases (bone, liver, lung, central nervous system, other), present anticancer treatment, and medications taken in the last 24 h. The Karnofsky Performance Status (KPS) scale will be used to assess performance status [34]. In addition, history and duration of opioid consumption will be obtained. Clinical chemistry results (hemoglobin, creatinine, C-reactive protein, albumin, and bilirubin) will be obtained from the medical record. Results collected less than 7 days before inclusion are considered valid.

The following populations will be considered for the analyses: (1) intention-to-treat population: all randomized patients regardless of protocol adherence; (2) per-protocol population: includes all subjects who have completed the study medication. A safety analysis will be performed, which includes all subjects who have received at least one dose of study medication. Subjects who withdraw from the study will be included in the safety analysis. The main statistical analysis will be performed when the target number of 100 patients has been recruited. Baseline characteristics will be summarized by mean and standard deviations (SDs) for continuous variables, and counts and percentages for categorical variables. The 11-point NRS for sleep quality is regarded as continuous. Comparison of means will be performed with the Student’s *t* test for continuous variables. The study follows the Consolidated Standards of Reporting Trials (CONSORT) recommendations for reporting RCTs. A *p* value of ≤0.05 will be considered statistically significant when comparing the two groups for the primary endpoint or the two secondary endpoints. Statistical analyses will be performed with SPSS version 25, IBM Statistics, Chicago, IL, USA.

### Trial procedures

**Fig. 3:**
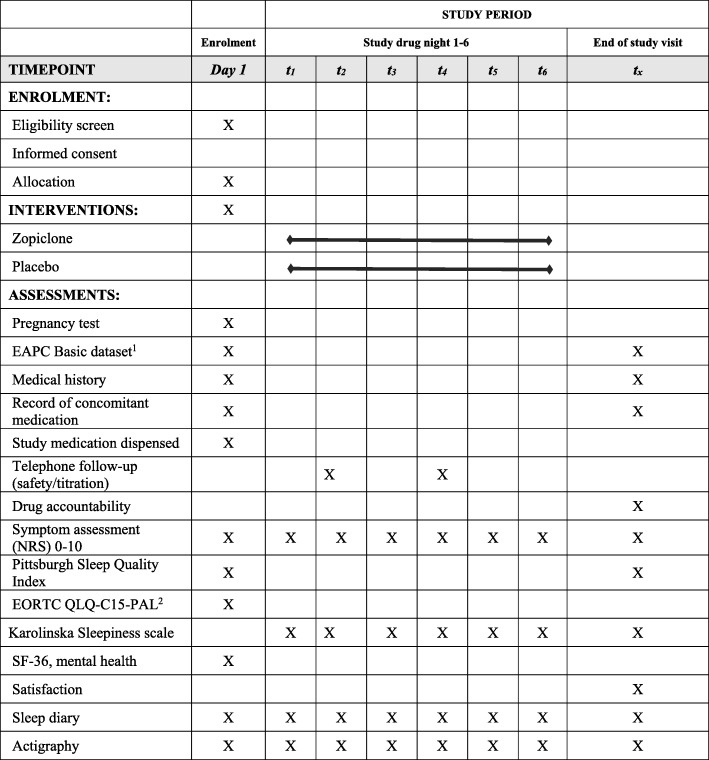
Study process schedule, according to the Standard Protocol Items: Recommendations for Interventional Trials (SPIRIT) guidelines

The SPIRIT schedule (Fig. 3) illustrates the schedule of the trial procedures from baseline to end of study.

### Patient-reported outcome measures

#### The European Association for Palliative Care (EAPC) basic dataset

This questionnaire includes assessment of symptoms such as pain, tiredness, drowsiness, nausea, lack of appetite, shortness of breath, depression, anxiety, wellbeing, sleep, constipation, and vomiting [35]. Patient-reported sleep quality is assessed on an NRS of 0–10, where 0 = Best sleep and 10 = Worst possible sleep.

#### The European Organisation for Research and Treatment of Cancer Core Quality of Life Questionnaire for palliative cancer patients (EORTC QLQ-C15-PAL)

This questionnaire for quality of life (QOL) in patients on palliative cancer care contains two functional scales (physical, emotional), two symptom scales (fatigue, pain), five single items (dyspnea, insomnia, appetite loss, nausea, constipation), and one item on global QOL. All items except global QOL are scored on a 4-point categorical scale covering the past week. There is one 7-point question on overall QOL [36].

#### Pittsburgh Sleep Quality Index (PSQI)

The PSQI is a self-rated questionnaire for evaluation of subjective sleep quality over the previous month [37]. This trial will use a version of the questionnaire for the assessment of sleep quality during the past week. The PSQI consists of 19 questions, which assess seven sleep components: subjective sleep quality, sleep latency, sleep duration, habitual sleep efficiency, sleep disturbances, use of sleep medications, and daytime dysfunction. The seven components, which are scored on a 0–3 scale, are summed to yield a global PSQI score between 0 and 21; higher scores indicate worse sleep quality [37].

#### Karolinska Sleepiness Scale (KSS)

The KSS is a 9-point Likert scale based on a self-reported, subjective assessment of the patient’s level of drowsiness at the time [38]. The questionnaire, with nine options ranging from “Very alert” to “Very sleepy, fighting sleep, difficulty staying awake” and including “Neither sleepy nor alert” and “Sleepy but no effort to remain awake” is scored from 1 to 9.

#### Health-related quality of life (HRQOL): mental health

HRQOL is assessed by the Short Form-36 (SF-36) questionnaire. The SF-36 is grouped into eight multi-item scales [39]. This trial will use five questions from the mental health scale.

#### Satisfaction

At the end of treatment, the patient will be asked a question on satisfaction: “How satisfied are/were you with the effect of the sleep medication?” Satisfaction with the use of sleep medication will be coded as either “not satisfied” (i.e., not at all satisfied, dissatisfied, and neither satisfied nor dissatisfied) or “satisfied” (i.e., satisfied and very satisfied) [40].

#### Sleep diary

The sleep diary is a subjective daily report of sleep and sleep disturbances, typically completed when getting up in the morning, to provide an estimate of the previous night’s sleep [41]. The sleep diary includes sleep onset latency (SOL), number of awakenings (NWAK), wake after sleep onset (WASO), terminal wakefulness (TWAK), total sleep time (TST), final awakening time, and sleep efficiency (SE). The sleep diary will be adapted according to the consensus recommendation for sleep diaries [42]. The trial will use the Norwegian version of the sleep diary published by the Norwegian Competence Center for Sleep Disorders, Haukeland University Hospital [43].

### Objective measure of sleep

*Actigraphy* is a method of assessment that distinguishes between wakefulness and sleep from the presence or absence of limb movement [44]. The Actiwatch is a small wristwatch-sized device which is used to assess sleep-wake cycles [45]. It has been demonstrated that actigraphy sleep measures are sensitive enough to detect changes related to drug interventions [45]. In this trial, Actiwatch 2 (Philips Respironics, Inc., Murrysville, PA, USA) will be used to collect actigraphy data. Sleep will be measured in one night without study treatment, and then the actigraphy will monitor sleep continuously during the 6 nights in the treatment period (nights 1–6). Data will be analyzed with Actiwatch Spectrum and the latest version of Actiware software. The medium sensitivity setting will be used with an epoch length (i.e., the period of time that the actigraphy data is averaged) of 30 s [46].

## Discussion

To improve palliative care, evidenced-based knowledge on the effects of therapeutic interventions is needed [47]. This randomized, double-blind, placebo-controlled, parallel-group, multicenter, clinical trial will provide important evidence concerning the short-time effectiveness of zopiclone for treatment of insomnia in palliative care. Results from this study of insomnia in patients with advanced cancer will inform clinical decision-making and provide guidance for treatment of sleep disturbance in these patients.

Recruiting patients with metastatic cancer is a major challenge, and research that involves patients with advanced cancer creates several ethical challenges. These patients are especially vulnerable and are at risk of several symptoms caused by the cancer disease and by short-time side effects of the therapy. It is important to recognize that the risks associated with research should not exceed the risks associated with usual care [48]. The patients included in this trial are exposed to some potential adverse effects from zopiclone. The risk is similar to that for sleep medications routinely administered to patients with sleep disturbance. It is possible that patients randomized to the control arm (placebo) may miss a beneficial therapy for 6 nights. However, today there is a clinical equipoise, and the best method to establish if zopiclone actually benefits or harms is a randomized placebo-controlled trial. Note also that placebos are frequently effective and can be as beneficial as supposedly active treatments [49]. Hence, the study is designed and will be performed for the best of the patients’ interest. Frequent consultations by phone with the patient during the treatment period will reveal if study drugs should be discontinued.

The primary endpoint in this clinical trial is patient-reported sleep quality in the last study night (night 6). Patient-reported outcome measures (PROMs) are especially important in palliative care, where symptom control is the major goal of interventions [50]. Research on insomnia management has primarily focused on specific sleep variables related to time intervals such as sleep initiation and sleep maintenance [51]. Recognizing the patients’ experience as more important in palliative care, we decided to use patient-reported sleep quality as more relevant to clinical practice.

In palliative care, many interventions are not supported by evidenced-based knowledge. This clinical trial assesses whether zopiclone, a frequently used sleep medication among palliative cancer care patients [24], is more effective than placebo in this cohort of patients.

### Organization

The sponsor is St. Olavs Hospital, Trondheim University Hospital, Trondheim, Norway. Study personnel at the European Palliative Care Research Centre (PRC), NTNU are responsible for the data management for the study. The principal investigator has insurance coverage for this study through membership in the Drug Liability Association, Norway. The patients included in the study will be covered by this insurance.

### Trial status

Participant recruitment is ongoing. Recruitment to the trial started in November 2016. In May 2017, it was decided to accept that both patients treated with an opioid and those not using an opioid can be included in the clinical trial. For this reason, the inclusion criterion “Regularly scheduled oral, subcutaneous, transdermal, or intravenous opioid treatment dose corresponding to step III at the WHO pain ladder with a stable dose for at least 2 days” was deleted from the protocol. The Regional Committee for Medical and Health Research Ethics, Norway approved this amendment in June 2017. The estimated date of completed recruitment is December 2019.

## Additional file

Additional file 1: SPIRIT 2013 checklist: recommended items to address in a clinical trial protocol and related documents. (PDF 97 kb)

## Abbreviations

AE: Adverse event
CBT-I: Cognitive behavioral therapy for insomnia
IMP: Investigational medicinal product
KPS: Karnofsky Performance Status
MCID: Minimal clinical important difference
NRS: Numerical rating scale
NWAK: Number of awakenings
PROM: Patient-reported outcome measure
RCT: Randomized controlled trial
SAE: Serious adverse event
SE: Sleep efficiency
SOL: Sleep onset latency
TST: Total sleep time
TWAK: Terminal wakefulness
WASO: Wake after sleep onset
